# Evaluation of thrombolysis by using ultrasonic imaging: an *in vitro* study

**DOI:** 10.1038/srep11669

**Published:** 2015-07-01

**Authors:** Jui Fang, Po-Hsiang Tsui

**Affiliations:** 1 Ph.D. Program in Biomedical Engineering, College of Engineering, Chang Gung University, Taoyuan, Taiwan; 2 Department of Medical Imaging and Radiological Sciences, College of Medicine, Chang Gung University, Taoyuan, Taiwan; 3 Medical Imaging Research Center, Institute for Radiological Research, Chang Gung University and Chang Gung Memorial Hospital, Taoyuan, Taiwan; 4 Department of Electrical Engineering, Chang Gung University, Taoyuan, Taiwan

## Abstract

The hematocrit of a thrombus is a key factor associated with the susceptibility to thrombolysis. Ultrasonic imaging is currently the first-line screening tool for thrombus examinations. Different hematocrits result in different acoustical structures of thrombi, which alter the behavior of ultrasonic backscattering. This study explored the relationships among thrombolytic efficiencies, hematocrits, and ultrasonic parameters (the echo intensity and backscattered statistics). Porcine thrombi with different hematocrits, ranging from 0% to 50%, were induced *in vitro*. An ultrasonic scanner was used to scan thrombi and acquire raw image data for B-mode (echo intensity measurements) and Nakagami imaging (backscattered statistics analysis). Experiments on thrombolysis were performed using urokinase to explore the effect of the hematocrit on thrombolytic efficiency. Results showed that the weight loss ratio of thrombi exponentially decreased as the hematocrit increased from 0% to 50%. Compared with the echo intensity obtained from the conventional B-scan, the Nakagami parameter predicts the weight loss ratio, increasing from 0.6 to 1.2 as the weight loss ratio decreased from 0.67 to 0.26. The current findings suggest that using Nakagami imaging characterizing thrombi provides information of backscattered statistics, which may be associated with the thrombolytic efficiency.

Thrombi represent a critical problem in modern society because they are associated with arterial occlusion (myocardial infarction and stroke) and venous thromboembolism (deep-vein thrombosis and pulmonary embolism). Each year, approximately 1.8 million people in the United States experience acute myocardial infarction and stroke^1^,^2^. The estimated annual incidence of venous thromboembolism in the United States is more than 250 thousand, and more than 100 thousand patients die because of pulmonary embolism^3^,^4^. Thrombolytic therapy with thrombolytic agents has gained worldwide recognition for treating myocardial infarction^5^,^6^ and ischemic stroke^7^. Thrombolytic agents are also valuable for treating hemodynamically significant pulmonary emboli^8^,^9^. Because thrombolytic therapy carries the risk of bleeding^1^,^10^, assessing the susceptibility of thrombi to lysis before treatment is vital.

Thrombi are primarily composed of red blood cells (RBCs) and platelets entrapped in a fibrin mesh^11^,^12^. The fibrin structure affects thrombolytic susceptibility because it determines the pore size and fiber diameter. Thrombi with large pore sizes and thick fibers are susceptible to lysis^13^,^14^,^15^,^16^,^17^,^18^. The volume percentages of RBCs in the thrombi (hematocrit) also influence lysability. Several previous studies have indicated that a high RBC concentration in a blood clot confers lytic resistance because of the decreasing fiber diameter and pore size^19^,^20^. Contradictory evidence was observed in other studies reporting that the accumulation of RBCs in a thrombus may facilitate thrombolysis^21^,^22^. The hematocrit of a thrombus is a key factor associated with the susceptibility to thrombolysis; however, the exact mechanism through which the hematocrit affects thrombolysis remains undetermined. Noninvasively detecting the hematocrit of a thrombus before thrombolytic treatments will provide opportunities to predict the thrombolytic response for possible treatment planning.

In clinical situations, medical imaging techniques have been widely used to screen and examine thrombi. Among all possibilities, ultrasonic imaging is the first-line screening tool because of its nonionizing radiation, relatively simple signal processing, and real-time capability. The primary scatterers in a thrombus are RBCs, which are smaller than the wavelengths of ultrasound used clinically (approximately 2–10 MHz). Thus, the echoes received from a thrombus are actually formed by ultrasonic scattering between scatterers and the incident wave. It has been shown that the received backscattered signals can provide useful clues regarding tissue properties^23^,^24^. Ultrasonic backscattering and its coefficients or intensity-related parameters have been used in detecting different hematocrits^25^,^26^,^27^,^28^,^29^. Different hematocrits may result in different concentrations and arrangements of scatterers (RBCs), causing the behavior of backscattered ultrasonic signals and the corresponding statistical distributions to depend on the acoustical structures of scatterers in the thrombi. Hence, analyzing the statistical distribution of backscattered ultrasonic signals may also be a potential method for differentiating thrombi on the basis of hematocrits.

To investigate the feasibility of using ultrasonic imaging in the pretreatment evaluation of thrombi, this study (i) examined ultrasonic parameters (the echo intensity and backscattered statistics) of thrombi as a function of the hematocrit and (ii) analyzed the relationships among thrombolytic efficiencies, hematocrits, and ultrasonic parameters. The following section demonstrates that susceptibility to thrombolysis depends on the hematocrit, which can be quantified using the echo intensity and backscattered statistics. The proposed strategy has potential in the pretreatment evaluation of thrombi in the future.

## Results

**Figure 1:**
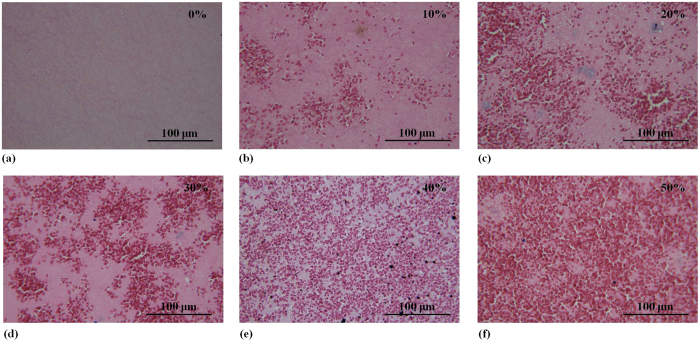
Hematoxylin and eosin-stained images of thrombi with different hematocrits ranging from 0% to 50%. With an increasing hematocrit, the number of red blood cells increased in the background of the thrombus.

**Figure 2:**
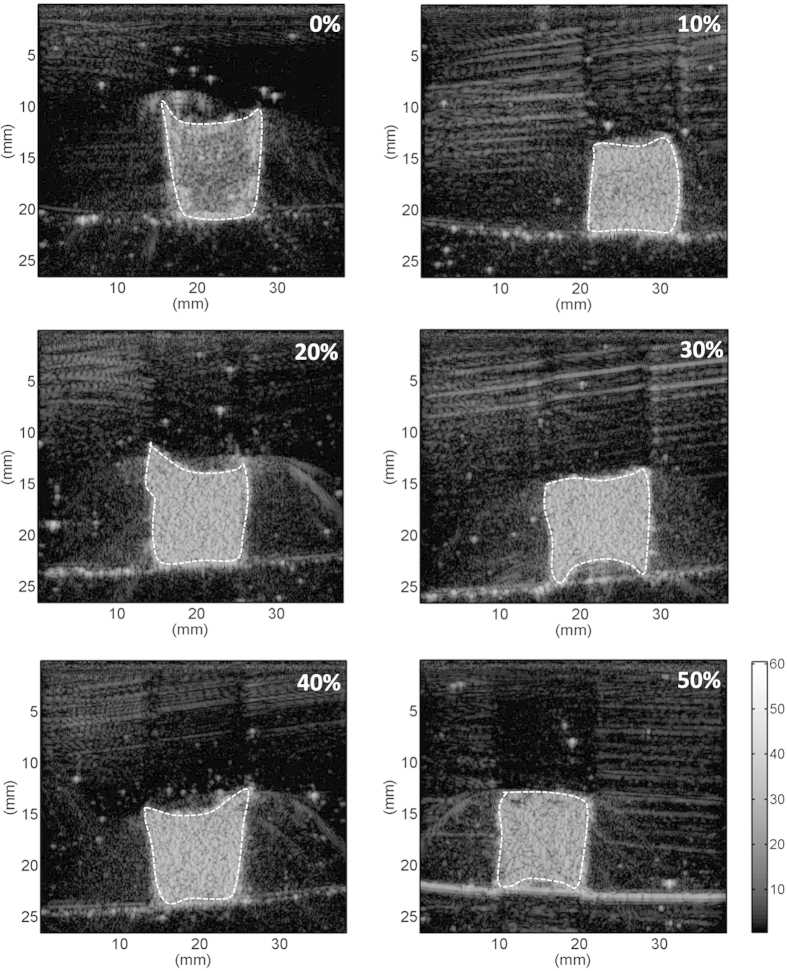
Ultrasonic B-mode images of thrombi with different hematocrits. The brightness of the B-mode images increased as the hematocrit increased from 0% to a maximum of 20%. Beyond the hematocrit of 20%, the brightness of the B-mode images decreased slightly. Whole blood samples were liquid; hence, no images are available.

**Figure 3:**
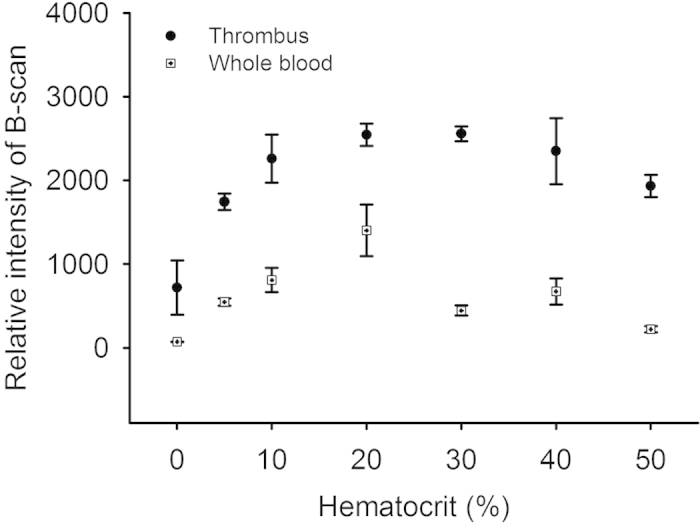
Echo intensities of whole blood and thrombi as a function of the hematocrit. The average echo intensity obtained from whole blood increased from 70.96 to 1401.52 when the hematocrit increased from 0% to 20%, and then decreased to 221 when the hematocrit increased to 50%. The intensity measured from thrombi also increased to a maximal value of 2546.15 at a hematocrit of 20% and decreased to 1931.93 when the hematocrit increased to 50%.

**Figure 4:**
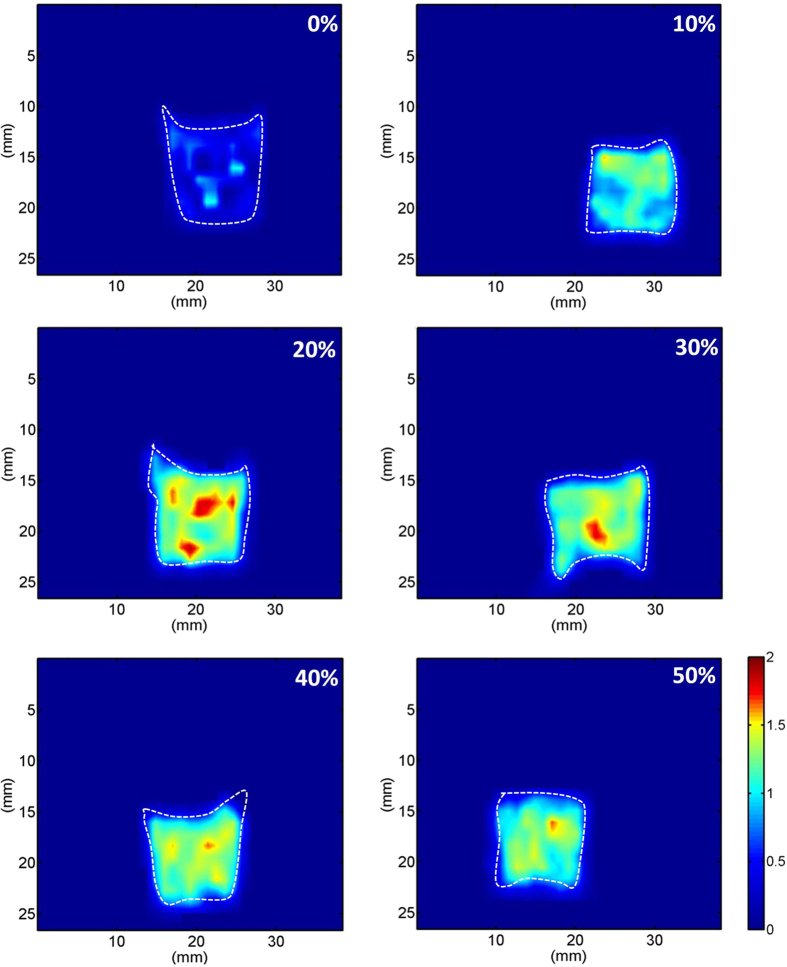
Nakagami images of thrombi with different hematocrits. The shading of the Nakagami image of the thrombi increased as the hematocrit increased from 0% to a maximum of 20%; however, beyond the hematocrit of 20%, the shading of the Nakagami image of the thrombi decreased slightly.

**Figure 5:**
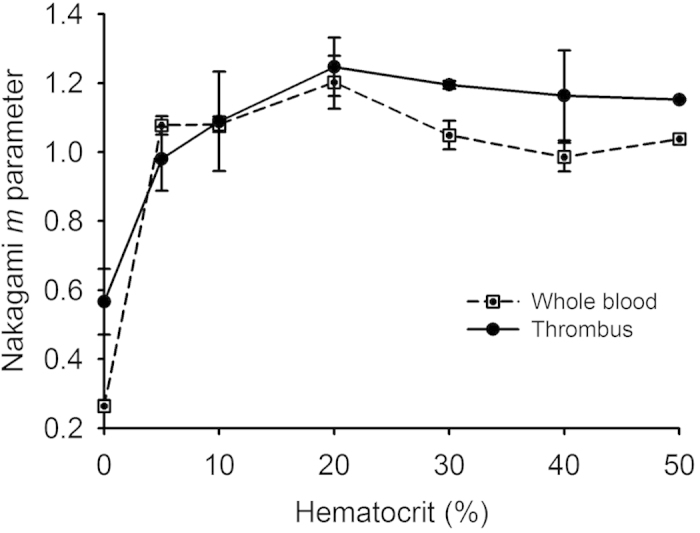
Nakagami parameters measured from whole blood and thrombi as a function of the hematocrit. The pair of Nakagami parameters obtained from the whole blood and thrombi (*m*_blood_, *m*_thrombus_) varied from 0.26 and 0.59 to 1.20 and 1.25, respectively, when the hematocrit increased from 0% to 20%, and slightly decreased to 1.03 and 1.15 when the hematocrit increased to 50%.

**Figure 6:**
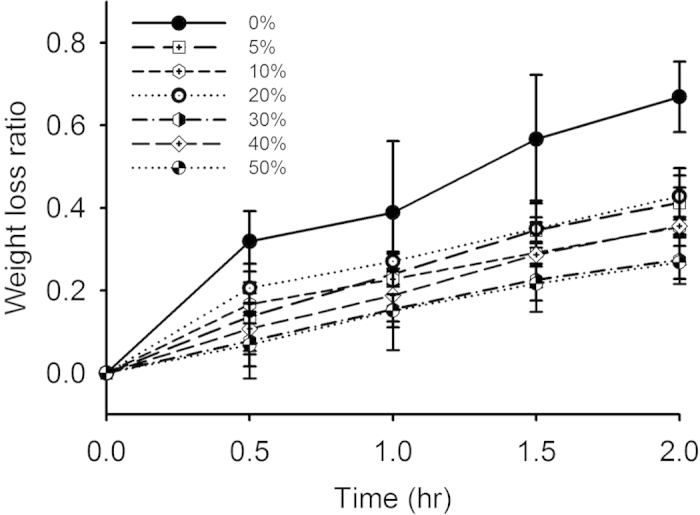
Weight loss ratios of thrombi with different hematocrits as a function of the lysis time. The weight loss ratio was approximately proportional to the lysis time for each hematocrit. The largest ratio of weight loss was observed at a hematocrit of 0% (%: the volume percentage of RBCs in a thrombus).

**Figure 7:**
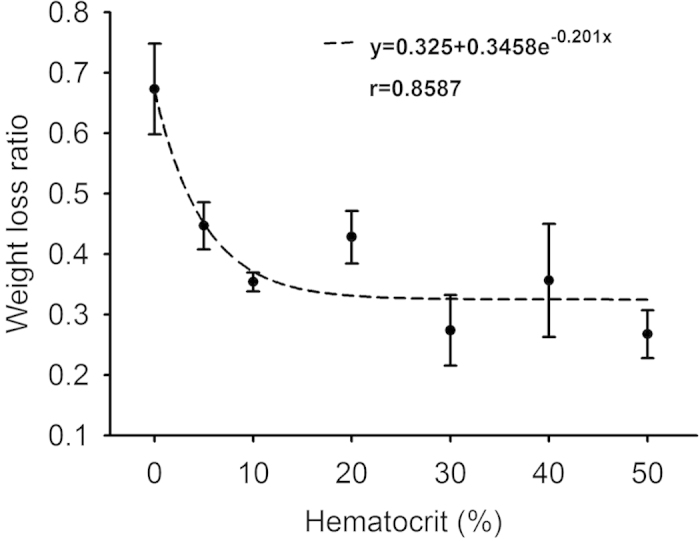
Curve of the weight loss ratio as a function of the hematocrit measured after thrombolysis for 2 h. The weight loss ratio exponentially decreased from approximately 0.67 to 0.26 as the hematocrit increased from 0% to 50%. An exponential fitting suggests that the weight loss ratio is an exponentially decreasing function of the hematocrit (correlation coefficient *r* = 0.86).

**Figure 8:**
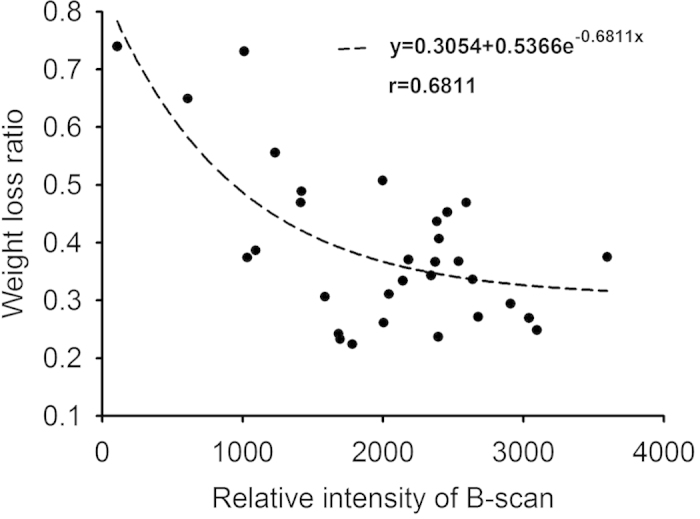
Weight loss ratio as a function of the echo intensity obtained from B-scan imaging. The weight loss ratio of a thrombus exponentially decreased from 0.7 to 0.3 as the intensity increased. The correlation coefficient obtained using the exponential decay function between the weight loss ratio and echo intensity was 0.68.

**Figure 9:**
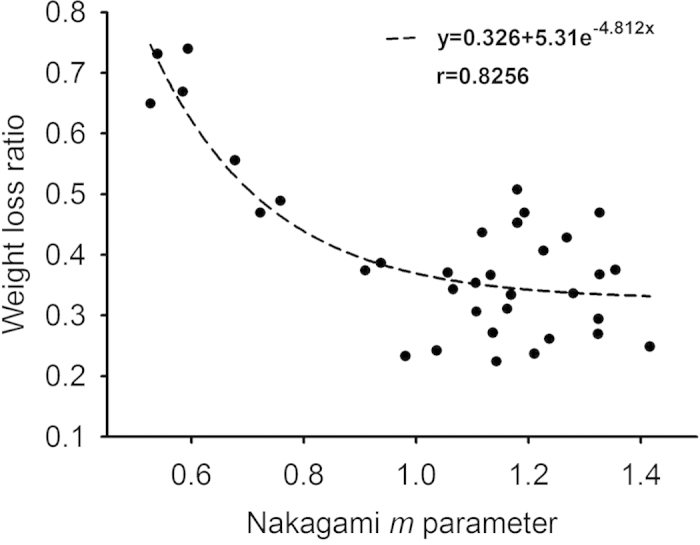
Weight loss ratio as a function of the Nakagami parameter obtained from Nakagami imaging. The weight loss ratio of thrombi exponentially decreased from 0.7 to 0.3 as the Nakagami parameter increased. Compared with the echo intensity, the Nakagami parameter has a higher correlation with the weight loss ratio (correlation coefficient = 0.83).

Figure 1 shows hematoxylin and eosin (H&E)-stained images prepared from thrombus samples with various hematocrits. With an increasing hematocrit, the RBC number increases in the background of the thrombus. Figure 2 shows ultrasonic B-mode images obtained from thrombi with different hematocrits. The average echo intensities obtained from whole blood and thrombi increased with the hematocrit and then decreased when the hematocrit was >20%, as shown in Fig. 3. Similar to the B-scan, the Nakagami images of thrombi appeared brighter with an increasing hematocrit, as shown in Fig. 4. This finding is supported by Fig. 5, which shows that the Nakagami parameters obtained from the whole blood and thrombi increased with the hematocrit and slightly decreased when the hematocrit was >20%. Figure 6 shows that the weight loss ratio was approximately proportional to the lysis time for each hematocrit, and Fig. 7 further indicates that thrombi with lower hematocrits have higher weight loss ratios. An exponential decay function with an equation form *y* *=* *y*_*0*_ + *ae*^*−bx*^ was used to fit the data in Fig. 7; the result suggests that the weight loss ratio exponentially decreases with the hematocrit (correlation coefficient *r* = 0.86; probability value *p* <0.01). The weight loss ratio of thrombi was found to decrease exponentially with an increasing intensity (*r* = 0.68; *p* < 0.01) and Nakagami parameter (*r* = 0.83; *p* < 0.01) (Figs 8 and 9). The results show that thrombi with lower intensities and Nakagami parameters have higher thrombolytic efficiencies.

## Discussion

### Significance of this study

The experimental findings are summarized as follows: (i) the echo intensity and Nakagami parameter can differentiate various hematocrits in the range of 0% to 20%; (ii) a thrombus with a low hematocrit is more sensitive to treatment with a thrombolytic agent because of a high weight loss ratio; (iii) the weight loss ratio of a thrombus following treatment with a thrombolytic agent depends on the measures of the echo intensity and Nakagami parameter. This study is the first to demonstrate that parameters of ultrasonic backscattering (measured before thrombolytic treatments) may reflect the thrombolytic efficiency.

### Effect of the hematocrit on the echo intensity

The trend of the echo intensity as a function of the hematocrit obtained from whole blood (Fig. 3) was consistent with the findings in previous studies on blood^27^,^30^. The echo intensities of thrombi are higher than those of whole blood. The differences in echogenicity between blood and thrombi may be contributed by fibrin fibers, which act as stronger scatterers (compared with RBCs) to increase the strength of ultrasonic backscattering measured from thrombi.

### Effect of the hematocrit on backscattered statistics

The behaviors of the Nakagami parameter as a function of the hematocrit of whole blood and thrombi were similar (Fig. 5). The Nakagami parameters measured from thrombi were greater than those measured from whole blood, although the difference in the Nakagami parameter between whole blood and thrombi was not as significant as that in the echo intensity. The Nakagami parameter is a shape parameter, which is less affected by the signal amplitude and mainly depends on the statistical distribution of backscattered data. Therefore, the effect of the hematocrit on the Nakagami parameters of whole blood and thrombi should be discussed by considering the arrangements and concentrations of scatterers in the scattering medium.

In whole blood, RBCs are the primary scatterers that determine the statistical distribution of backscattered data. It is well known that backscattered statistics obey the Rayleigh distribution when the numbers of scatterers in the resolution cell of the ultrasonic transducer are higher than 10^31^,^32^,^33^. The resolution cell used in our experiments was at a level of cubed millimeters, and the mean volume of a RBC is approximately 63 μm^3^ for pigs^30^. Except at a hematocrit of 0%, the whole blood samples had many RBCs randomly distributed in the resolution cell, causing backscattered statistics to follow the Rayleigh distribution and the corresponding Nakagami parameters to approximate 1. A high number of RBCs in whole blood may still have an aggregation effect to some degree; hence, the Nakagami parameters corresponding to the hematocrits larger than 20% varied between 1 and 1.2. Similar results have been obtained in previous studies^27^,^34^.

At least two types of scatterers should be considered in thrombi: RBCs and fibrin fibers. A hematocrit of 0% indicates that no RBCs in a thrombus contribute echoes. In this condition, the fibrin fibers, which exhibit diverse shapes, sizes, and scatterer spacings, become the dominant scatterers and generate a scattering environment with a high degree of variances in the scattering cross sections of scatterers. Hence, the backscattered statistics tend to follow a pre-Rayleigh distribution in accordance with the Nakagami parameters lower than 1. With an increasing hematocrit, RBCs gradually replace the fibrin fibers to become the primary scatterers dominating the formation of backscattered signals, changing the acoustical structure of a thrombus from a relatively nonuniform medium (a mixture of fibrin fibers and RBCs) to a more uniform arrangement of fibers embedded with a large number of randomly distributed scatterers^21^. As long as the acoustical structure of a thrombus is based on a high number of scatterers, the backscattered statistics remain close to the Rayleigh distribution, and the Nakagami parameter is approximately 1.

### Effect of the hematocrit on thrombolysis

The current results demonstrated that the susceptibility of a thrombus to lysis depends on the hematocrit. The weight loss ratio of a thrombus as a function of the lysis time for each hematocrit follows an approximately linear relationship (Fig. 6). This result conformed to the distance run by the fibrin boundary layer of a thrombolytic agent^19^ and the fiber lysis rate according to plasma thrombi (thrombi with 0% hematocrit)^15^. The overall thrombolytic efficiency after 2-h lysis indicated that RBCs increase lytic resistance. As shown in Fig. 7, the weight loss ratios of thrombi decreased from 0.67 to 0.26 with increasing the hematocrit from 0% – 30%, and those corresponding to the hematocrits >30% varied in a limited range between 0.25 and 0.35. This may be explained by two reasons. First, the occlusion of pores in fibrin networks by RBCs reduces the pore size^20^, influencing permeability^35^,^36^, which is the most crucial mode of enzyme transport from the outside to the inside of thrombi^35^,^37^. Second, RBC fibrinogen receptors cause fibers to thin^19^,^38^ and, thus, have fewer binding sites for thrombolytic agents, limited access for thrombolytic agents to the binding sites, or a combination of both^13^. This reduces the interaction between fibrin fibers and thrombolytic agents, and hence reduces the thrombolytic efficiency.

### Potential of backscattered statistics in thrombolysis evaluation

Note that clinical thrombolytic treatment carries the hazard of secondary hemorrhage^1^,^10^, and thus it would be valuable to know in advance whether the offending thrombus is susceptible or inherently resistant to thrombolytic treatment. Our proposed concept, based on the analysis of ultrasonic backscattering statistics, may enable evaluating the outcome of thrombolysis before treatment. The results in Figs 8 and 9 indicated that the weight loss ratio of a thrombus following treatment with thrombolytic agents appears to be an exponential decay function of the ultrasonic parameters (the echo intensity and Nakagami parameter). In particular, the Nakagami parameter, based on backscattered statistics, has a stronger correlation with the weight loss ratio of thrombi according to the correlation coefficient obtained from curve fitting. The experimental findings based on the current data suggest that ultrasound Nakagami imaging can characterize thrombi to provide information of backscattered statistics, which may be associated with the thrombolytic efficiency.

#### Future work

Some concerns must be clarified for future clinical applications: (i) the properties of thrombi obtained in *in vitro* and *in vivo* environments are different. The spatial distribution of RBCs in thrombi produced *in vivo* is relatively nonuniform compared to that produced in *in vitro* samples. Unlike *in vitro* thrombi mainly composed of uniform fibrin and RBCs, the structures of *in vivo* thrombi also include platelets, fibrin-platelet patterns^39^, or platelet aggregations^40^. The distribution of platelets in a thrombus is actually an important factor in thrombolysis. For instance, the laminations of coalescent platelets mixed with the fibrin network (known as Zahn lines^41^,^42^) in *in vivo* thrombi may result in a stronger resistance to thrombolysis^43^. For these reasons, the performance of using Nakagami imaging to evaluate the lysis of thrombus *in vivo* should be investigated using animal models. (ii) The thrombus age is also a factor that affects the efficiency of thrombolysis. Thrombi with different ages have different degrees of fibrin cross-bridging. It has been shown that aged (retracted) thrombi have a stronger resistance to thrombolysis^44^,^45^. The dependencies of the Nakagami parameter on the weight loss ratios of thrombi with different ages should also be established. (iii) The susceptibility to thrombolysis depends not only on the thrombus composition but also on the flow and systemic factors. Flow dynamics may vary according to different vessel locations, velocities, diameters, and branching anatomies, causing the permeability of thrombi to change to influence the susceptibility to thrombolysis^16^,^35^,^46^. Systemic factors such as blood glucose and plasminogen activator levels may also influence the activity of thrombolytic agents^47^. The aforementioned concerns require clinical measurements to clarify.

## Conclusion

This study used an *in vitro* model to perform ultrasonic B-mode and Nakagami parametric imaging of thrombi with different hematocrits for comparison between the weight loss ratios of thrombi following treatment with thrombolytic agents and ultrasonic parameters. The experimental results demonstrated that the hematocrit of a thrombus affects the material structure and corresponding response to thrombolysis. Compared with the echo intensity obtained from the conventional B-scan, the Nakagami parameter estimated from ultrasonic Nakagami imaging improves the differentiation of various hematocrits and correlates well with the weight loss ratios of thrombi. The current findings suggest that using ultrasonic Nakagami imaging characterizing a thrombus provides information of backscattered statistics, which may be associated with the thrombolytic efficiency.

## Materials and Methods

**Figure 10:**
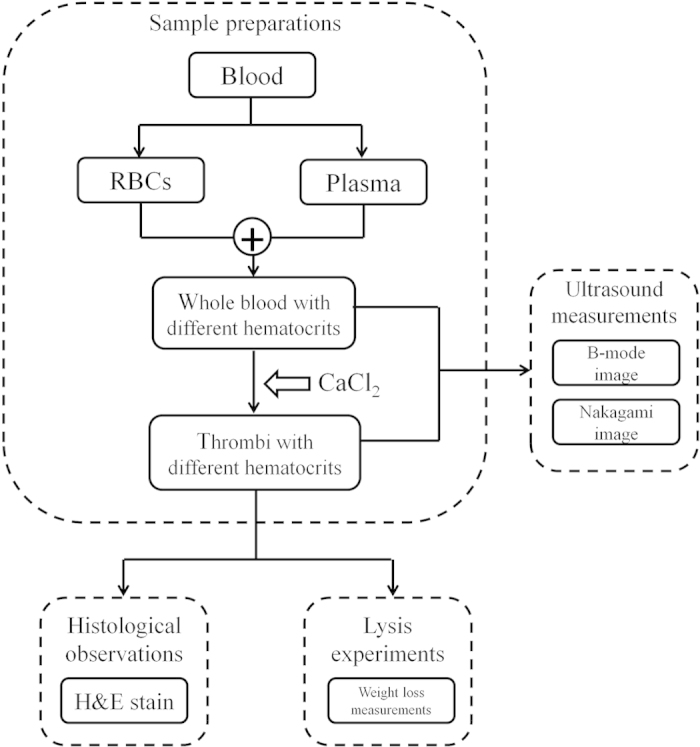
Flowchart of the experiments, including sample preparations, ultrasonic measurements, histological observations, and experiments on thrombolysis.

The experimental design is illustrated in Fig. 10 and includes sample preparations, ultrasonic measurements, histological observations, and experiments on thrombolysis.

### Sample preparations: whole blood

Fresh porcine blood containing a 15% acid citrate dextrose anticoagulant solution was collected from a local slaughterhouse, and no specific permission was required. Porcine blood is a suitable material for fundamental research because of its availability and biochemical similarity to human blood^48^. Ultrasonic backscattering depends on the dimensions of scatterers. Because the dimensions of porcine RBCs (63 μm^3^) are close to those of human RBCs (87 μm^3^)^30^, the generated backscattered echoes in porcine blood may behave like those of humans. The blood was passed through a sponge to filter out impurities and was discarded whenever coagulation was observed. The blood was centrifuged at room temperature for 15 min at 1500 rpm to separate RBCs from the plasma. Whole blood was prepared by adding RBCs to plasma to obtain hematocrits of 0%, 5%, 10%, 20%, 30%, 40%, and 50%. For each hematocrit, 10 samples were prepared (total *n* = 70).

### Sample preparations: thrombi

To induce thrombi *in vitro*, whole blood for each hematocrit was added to a container, which was placed on a stirrer to be stirred for 15 min and bathed in water at 37.3 °C for 20 min while being exposed to room air for equilibrating the gas content. Thrombi coagulation was initiated by adding 0.5 M CaCl_2_ at a ratio of 1:10^49^,^50^, and the coagulum was molded in a 6-mL syringe. The syringes were incubated at 37.3 °C for 3 h during coagulation. The average volume of thrombi was approximately 1 cm^3^. Ten animals were used to induce thrombi for each hematocrit (total *n* = 70).

### Ultrasonic measurements of whole blood and thrombi

**Figure 11:**
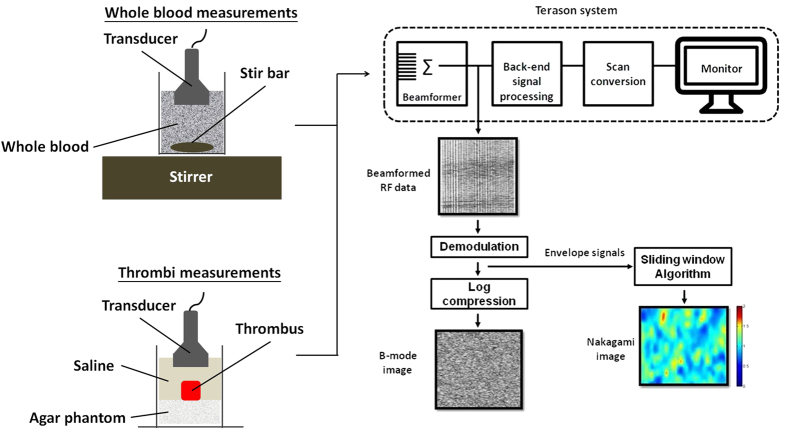
Illustration of how we performed ultrasonic measurements and analyzed the echo intensity and statistical distribution of backscattered signals.

Figure 11 explains how we performed ultrasonic measurements and analyzed the echo intensities and statistical distributions of backscattered signals. Prior to experiments on thrombi, evaluating whole blood (without fibrin structures) for echo intensities and backscattered statistics as a function of the hematocrit is necessary to clarify the effects of RBCs and fibrin structures on ultrasonic signals. In whole blood, rouleaux formation or RBC aggregation occurs, resulting in the nonuniform distribution of scatterers^51^. For whole blood measurements, an ultrasonic transducer was immersed in blood, which was stirred using a magnetic stirrer at a rotational speed of 200 rpm to randomly distribute RBCs in the plasma.

For thrombotic measurements, each thrombus sample was placed on an agar phantom in an acrylic case filled with a saline solution. An agar phantom was used as a bottom layer to prevent the overlap of strong reflection signals from the bottom of the case with backscattered signals from the thrombi.

Both whole blood and thrombus samples were imaged using a clinical ultrasonic scanner (Model 3000, Terason, Burlington, MA, USA) equipped with a 7.5-MHz linear array transducer (Model 10L5, Terason) to acquire raw beamformed radiofrequency (RF) data consisting of 256 scan lines of backscattered signals. The focal zone was adjusted to the center of the samples to reduce the effect of beam diffraction. The time gain compensation was not used. The pulse length of the transducer and the sampling rate of RF signals were 0.7 mm and 30 MHz, respectively. In total, five independent scans of each sample were performed. The system settings remained the same for all image data acquired.

For each image RF datum, each scan line was demodulated using the absolute value of Hilbert transform^52^ to construct an envelope image for ultrasonic B-mode (echo intensity calculation) and Nakagami imaging (backscattered statistics analysis).

### Ultrasonic B-mode and Nakagami imaging

The ultrasonic B-mode image was formed using a logarithm-compressed envelope image (dynamic range = 60 dB). Ultrasonic Nakagami imaging was used to measure the backscattered statistics. The Nakagami image is a well-established ultrasonic parametric image based on the Nakagami parameter *m* used for backscattered statistics analysis^53^,^54^,^55^,^56^,^57^,^58^,^59^. The Nakagami parameter is a shape parameter of the Nakagami statistical model and can be estimated as follows:
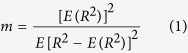
where *R* is the envelope of backscattered ultrasonic signals^60^. In general, the backscattered statistics of the envelopes received from biological tissues can be divided into three distribution types^61^,^62^: (i) the Rayleigh distribution caused by a high number of randomly distributed scatterers in the resolution cell of the transducer; (ii) the pre-Rayleigh distribution (with a phase lead compared to Rayleigh statistics) caused by a low scatterer concentration or the presence of scatterers in the resolution cell with randomly varying scattering cross sections with a high degree of variance; and (iii) the post-Rayleigh distribution (with a phase lag compared with Rayleigh statistics) caused by a resolution cell containing periodically located scatterers in addition to randomly distributed scatterers. A study showed that the variation of the Nakagami parameter from 0 to 1 corresponds to a change in the envelope statistics from pre-Rayleigh to Rayleigh distributions; Nakagami parameters higher than 1 indicate that the statistics of the backscattered signal conform to post-Rayleigh distributions^60^. Consequently, the Nakagami distribution is a general model for ultrasonic backscattering.

The details of the algorithm for ultrasonic Nakagami imaging can be found in previous studies^62^,^63^,^64^,^65^. In brief, the Nakagami image is based on the Nakagami parametric map, which is constructed using a square sliding window to process the envelope image (without logarithmic compression). This involves two main steps: (i) a square window within the envelope image is used to collect local backscattered envelopes for estimating the local Nakagami parameter, which is assigned as the new pixel located in the center of the window; and (ii) the window is allowed to move throughout the envelope image in one-pixel steps, and Step 1 is repeated to construct the map of the local Nakagami parameter. Previous studies have suggested that the side length of the square window that can simultaneously satisfy the image resolution and stable parameter estimation is three times the pulse length of the transducer^63^. In this study, we used a square window with a side length of 2.1 mm, corresponding to three times the transducer pulse length, to construct the Nakagami image.

### Image data analysis

For each sample, the average echo intensity and Nakagami parameter were calculated using the envelope and Nakagami images, respectively. A region of interest with a size of 5 × 5 mm^2^ was used to acquire the corresponding image pixel data for averaging. The curves of the relative intensity (i.e., envelope amplitude) and Nakagami parameter as functions of the hematocrit were plotted. Data are expressed as means ± standard deviation (SD).

### Histological observations

To confirm the change in the number of RBCs with an increasing hematocrit, each thrombus was processed using H&E staining. Each thrombus was immersed in a 10% formaldehyde-buffered solution for 24 h. Thrombi were then paraffin-embedded, cut into 5-μm sections, and stained with H&E. Histological images of whole thrombi (400 × magnification) were photographed using a Leica DM759 bright-field microscope with a color digital camera (Canon EOS600D).

### Experiments on thrombolysis

After ultrasonic measurements, experiments on thrombolysis were performed to examine the effect of the hematocrit on the thrombolytic efficiency. Each thrombus was placed in an acrylic case filled with a thrombolytic agent solution, which was incubated at 37.3 °C. The thrombolytic agent solution was prepared by dissolving commercial lyophilized urokinase powder (250000 IU, Green Cross Co. Ltd., Taipei, Taiwan) in 25 mL of normal saline, according to the manufacturer’s instructions. The thrombolysis efficiency obtained using urokinase is almost the same as that of other thrombolytic agents (e.g., streptokinase or tissue-type plasminogen activator)^66^,^67^. However, urokinase is suggested for *in vitro* tests only because of a poor fibrin specificity^1^. During experiments, the ratios of weight loss for thrombi with hematocrits were measured every 30 min to 2 h. The ratios of weight loss (data are expressed as means ± SD) as a function of time were plotted to evaluate the thrombolytic efficiencies at different hematocrits. Measuring weight loss is a simple experimental technique that is minimally susceptible to intersample variability^58^.

### Statistical analysis

According to observations on the relationships between the weight loss of a thrombus and ultrasonic parameters (the echo intensity and Nakagami parameter), nonlinear curve fitting in an equation of the form *y* *=* *y*_*0*_ + *ae*^*−bx*^ was carried out using the SigmaPlot software (Version 9.0, Systat Software, Inc., CA, USA) to calculate the correlation coefficient *r* and the probability value (*p*-value).

## Additional Information

**How to cite this article**: Fang, J. and Tsui, P.-H. Evaluation of thrombolysis by using ultrasonic imaging: an *in vitro* study. *Sci. Rep*. **5**, 11669; doi: 10.1038/srep11669 (2015).
